# Global land cover trajectories and transitions

**DOI:** 10.1038/s41598-021-92256-2

**Published:** 2021-06-17

**Authors:** Taher M. Radwan, G. Alan Blackburn, J. Duncan Whyatt, Peter M. Atkinson

**Affiliations:** 1 Lancaster Environment Centre, Lancaster University, Lancaster, LA1 4YQ UK; 2 Department of Soil and Water Sciences, Faculty of Agriculture (El-Shatby), Alexandria University, Alexandria, 21545 Egypt; 3 Institute of Geographic Sciences and Natural Resources Research, Chinese Academy of Sciences, Beijing, 100101 China; 4 Geography and Environmental Science, University of Southampton, Highfield, Southampton, SO17 1BJ UK

**Keywords:** Environmental sciences, Sustainability

## Abstract

Global land cover (LC) changes threaten sustainability and yet we lack a comprehensive understanding of the gains and losses of LC types, including the magnitudes, locations and timings of transitions. We used a novel, fine-resolution and temporally consistent satellite-derived dataset covering the entire Earth annually from 1992 to 2018 to quantify LC changes across a range of scales. At global and continental scales, the observed trajectories of change for most LC types were fairly smooth and consistent in direction through time. We show these observed trajectories in the context of error margins produced by extrapolating previously published accuracy metrics associated with the LC dataset. For many LC classes the observed changes were found to be within the error margins. However, an important exception was the increase in urban land, which was consistently larger than the error margins, and for which the LC transition was unidirectional. An advantage of analysing the global, fine spatial resolution LC time-series dataset is the ability to identify where and when LC changes have taken place on the Earth. We present LC change maps and trajectories that identify locations with high dynamism, and which pose significant sustainability challenges. We focused on forest loss and urban growth at the national scale, identifying the top 10 countries with the largest percentages of forest loss and urban growth globally. Crucially, we found that most of these ‘worst-case’ countries have stabilized their forest losses, although urban expansion was monotonic in all cases. These findings provide crucial information to support progress towards the UN’s SDGs.

## Introduction

In recent decades, the world has been impacted significantly by human-induced environmental changes from local to global scales^1^. In particular, anthropogenic land cover (LC) changes threaten the sustainability of ecosystem services^2^. Major LC changes include urbanisation^3^, agricultural land loss^4^, agricultural land expansion^5^, deforestation^6^, afforestation^7^ and desertification^8^. Such LC changes can have detrimental impacts on both environmental conditions^9^ (e.g. by inducing pollution and climate change) and human activities^10^ (e.g. by compromising food security and economic development). Therefore, with such a variety of forms of LC change and consequent impacts, there is a pressing need for rigorous and systematic monitoring and analysis of LC dynamics to inform research on the processes involved, and provide evidence to stakeholders and decision-makers across the globe to promote responsible actions^11^.

Land change science (LCS), plays a pivotal role in monitoring global environmental change and the sustainability of our planet’s resources^12^. The main goal of LCS is to understand both the magnitude and spatial extent of changes in LC over time^13^. Furthermore, LCS endeavours to identify the drivers of LC change, investigate the possible impacts and potential consequences of LC dynamics, propose better land use planning policies, and inform relevant decision-makers^1,13^. Consequently, this will help address many emerging environmental and societal challenges^11,13^. Within the context of LCS, maps of LC are valuable tools for presenting geospatial information for a wide range of environmental applications^14,15^. Satellite remote sensing is increasingly capable of generating LC maps at various spatial and temporal resolutions, appropriate for a variety of research objectives to support Earth observation^16,17^.

Over the past two decades, several remote-sensing based LC mapping projects have been established, operating at a variety of scales^17^. These projects have generated LC datasets for different time periods and spatial resolutions, with varying classification schemes^16^. Medium-to-fine spatial resolution remotely sensed data are often used at national or regional scales, to develop products such as CORINE Land Cover in Europe^18^ and the National Land Cover Database (NLCD) in the United States of America^19^. Coarser spatial resolution remotely sensed data are typically used to generate global LC products^16^ including the International Geosphere-Biosphere Program Data and Information System's LC dataset (IGBP-DISCover^20^), University of Maryland (UMD) Land Cover^21^, Global Land Cover (GLC) 2000^22^, GlobCover 2009^23^ and the Moderate Resolution Imaging Spectroradiometer (MODIS) collection 5 land cover type (MCD12Q1) dataset developed by NASA, with a spatial resolution of 500 m and global coverage annually from 2001 onwards^24^. The Finer Resolution Observation and Monitoring Global (FROM-GLC^25^) and GlobeLand30^26^ LC datasets cover the globe based on relatively finer spatial resolution Landsat images. However, the latter is only available for the years 2000 and 2010.

Recently, the European Space Agency’s Climate Change Initiative-Land Cover (ESA-CCI-LC^27^) dataset was released, which has a spatial resolution of 300 m and global coverage annually from 1992 to 2018. The value of this dataset has been demonstrated in several studies of specific types of environmental change at different scales^28–35^. However, the full capacity of this dataset to provide a comprehensive assessment of LC change trajectories and transitions at global and continental scales, has yet to be explored. In this study, we characterised and interpreted historical LC changes that have occurred across the entire globe and analysed the variability of LC dynamics between, and within, the Earth’s continents over the 27-year timeframe of the ESA-CCI-LC dataset. For each LC type, we quantified the total area that has been gained and lost over the study period and mapped the distribution of these changes. We analysed the trajectories of LC change throughout the study period and quantified the magnitude of the transitions between different combinations of LC types. These LC changes were considered in the context of error margins produced by extrapolating previously published accuracy metrics associated with the LC dataset.

## Results

### Gains and losses at the global scale

**Figure 1:**
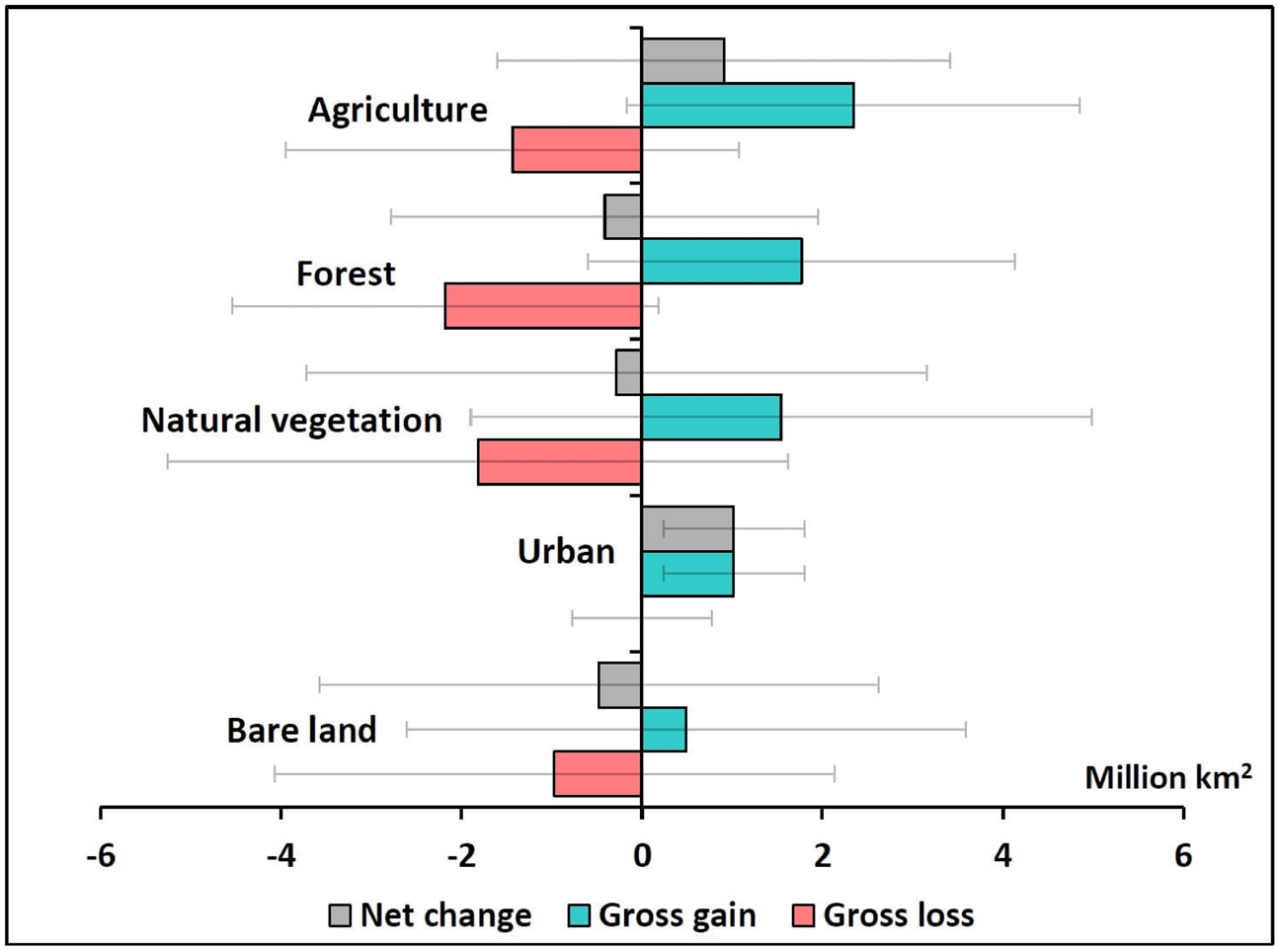
Total area of gains and losses of the different LC types across the globe between 1992 and 2018. Error bars represent the margin of error at the 95% confidence interval.

Total global gains and losses (gross and net) of the main LC types between 1992 and 2018 are shown in Fig. 1. All LC types, apart from urban, showed sizeable gross gains and losses, indicating that many areas of the globe experienced an expansion of these LC types while many other areas experienced a contraction. Most changes were smaller than the error margins associated with the ESA-CCI-LC dataset suggesting that there remains some uncertainty in determining the direction (positive or negative) of the net changes of these LC types at the global scale. The exception was urban, which showed a gross increase larger than the error margin and, in the absence of any gross loss, a sizeable net increase of 1.02 ± 0.78 million km^2^.

### Gains and losses at the continental scale

**Figure 2:**
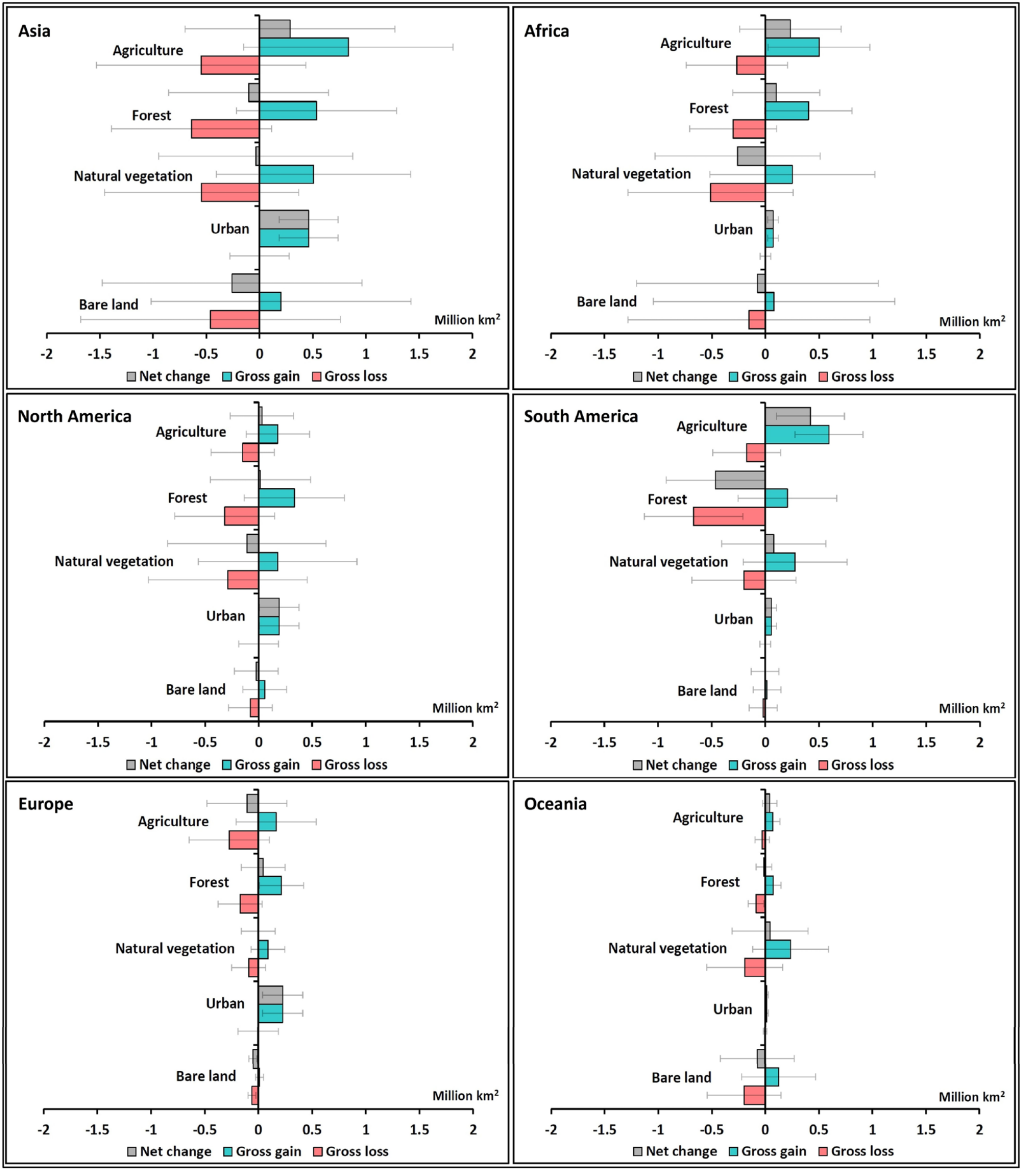
Total area of gains and losses of the different LC types in each continent between 1992 and 2018. Error bars represent the margin of error at the 95% confidence interval.

**Figure 3:**
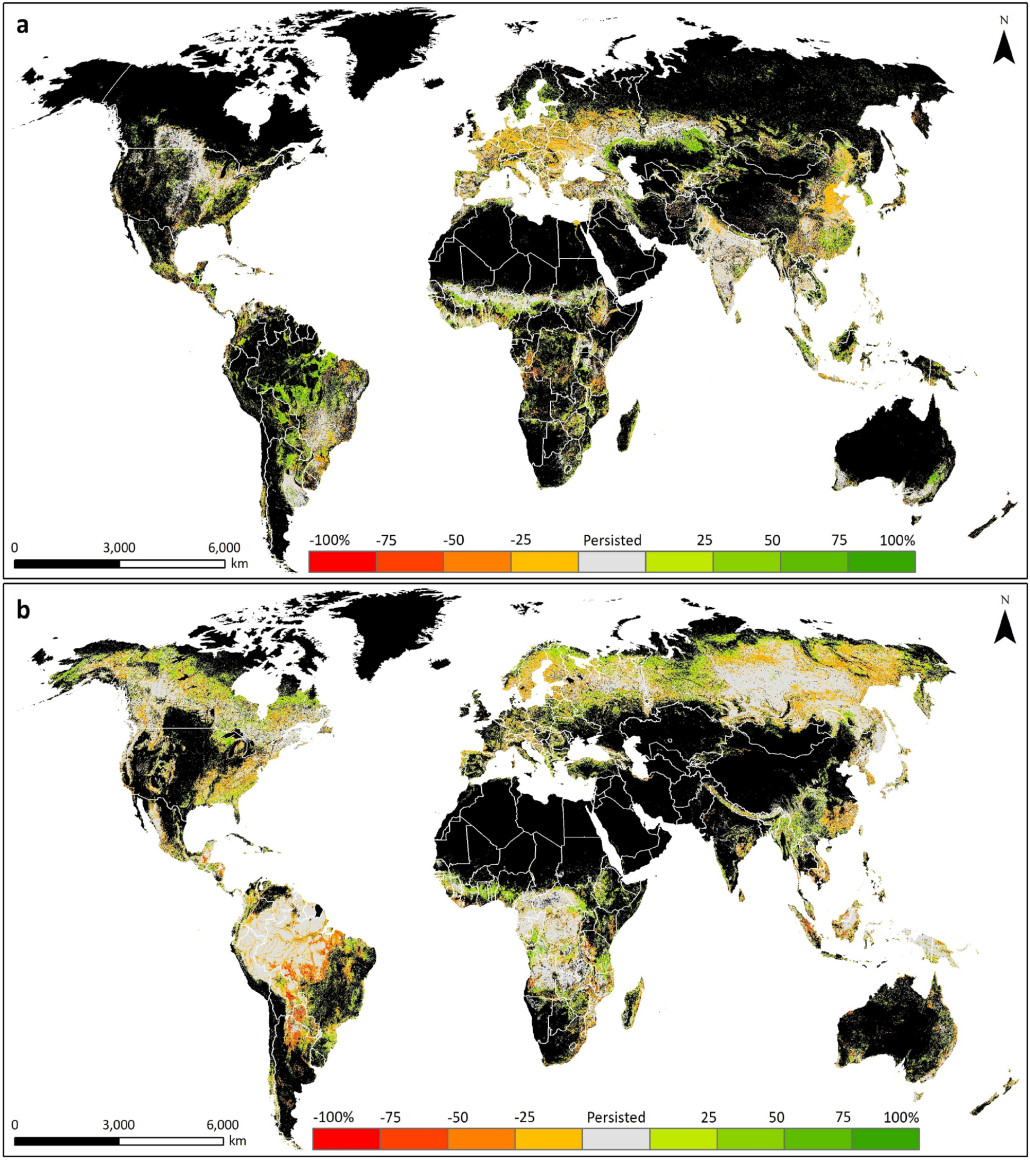
Spatial distribution of LC changes between 1992 and 2018. (**a**) agricultural land and (**b**) forest cover. The original data were aggregated to a 3 km spatial resolution for visualisation. Black areas are terrestrial zones where the LC type was absent in both 1992 and 2018. ArcGIS Desktop 10.5^36^ (https://desktop.arcgis.com/en/) was used to generate this map.

All continents showed substantial gross gains and losses in most LC types, with the largest changes in Asia, Africa and South America (Fig. 2, with data provided in Table S1 for clarity). For most LC types and continents, the changes were smaller than the empirical error margins associated with the ESA-CCI-LC dataset suggesting that it is not possible to determine the direction of the net changes. However, some changes were greater than the error margins. Notably, there was a net increase in urban in all continents, with Asia experiencing the largest net gain, contributing 45% of the global gain in urban area. Also, South America had a large net increase in agriculture and large net loss in forest. Maps of the spatial distribution of LC gains and losses (Fig. 3 and Fig. S1) show the variability between and within continents as well as identifying countries with high LC dynamism.

### Trajectories of LC types at the global scale

**Figure 4:**
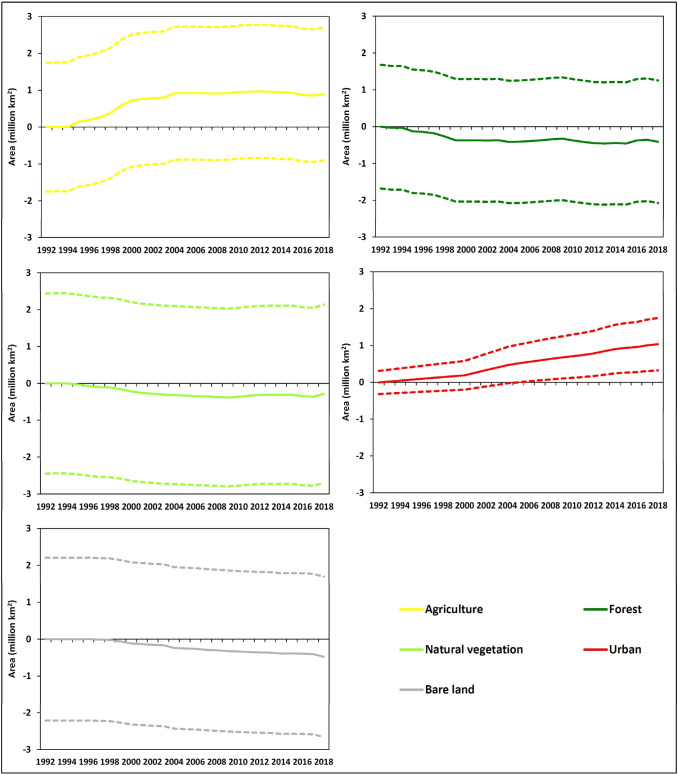
Time-series of the cumulative net change in total global area of each LC type between 1992 and 2018. The colours of the lines representing each LC type are consistent with Figs. 6 and 10. The dashed lines represent the upper and lower bounds of error at the 95% confidence interval.

The cumulative net changes in the total global area of each LC type between 1992 and 2018 are shown in Fig. 4. As suggested by the analysis of the overall net changes above, for most LC types the variability in area was within the error margins associated with ESA-CCE-LC product. However, the trajectories were reasonably smooth and consistent over the annual time-series. Hence, there is some evidence that, globally, forest and natural vegetation decreased more rapidly initially then stabilised, bare land was stable initially then decreased continuously, and agriculture increased more rapidly initially then stabilised. There exists clear evidence that urban increased continuously over time.

### Trajectories of LC types at the continental scale

**Figure 5:**
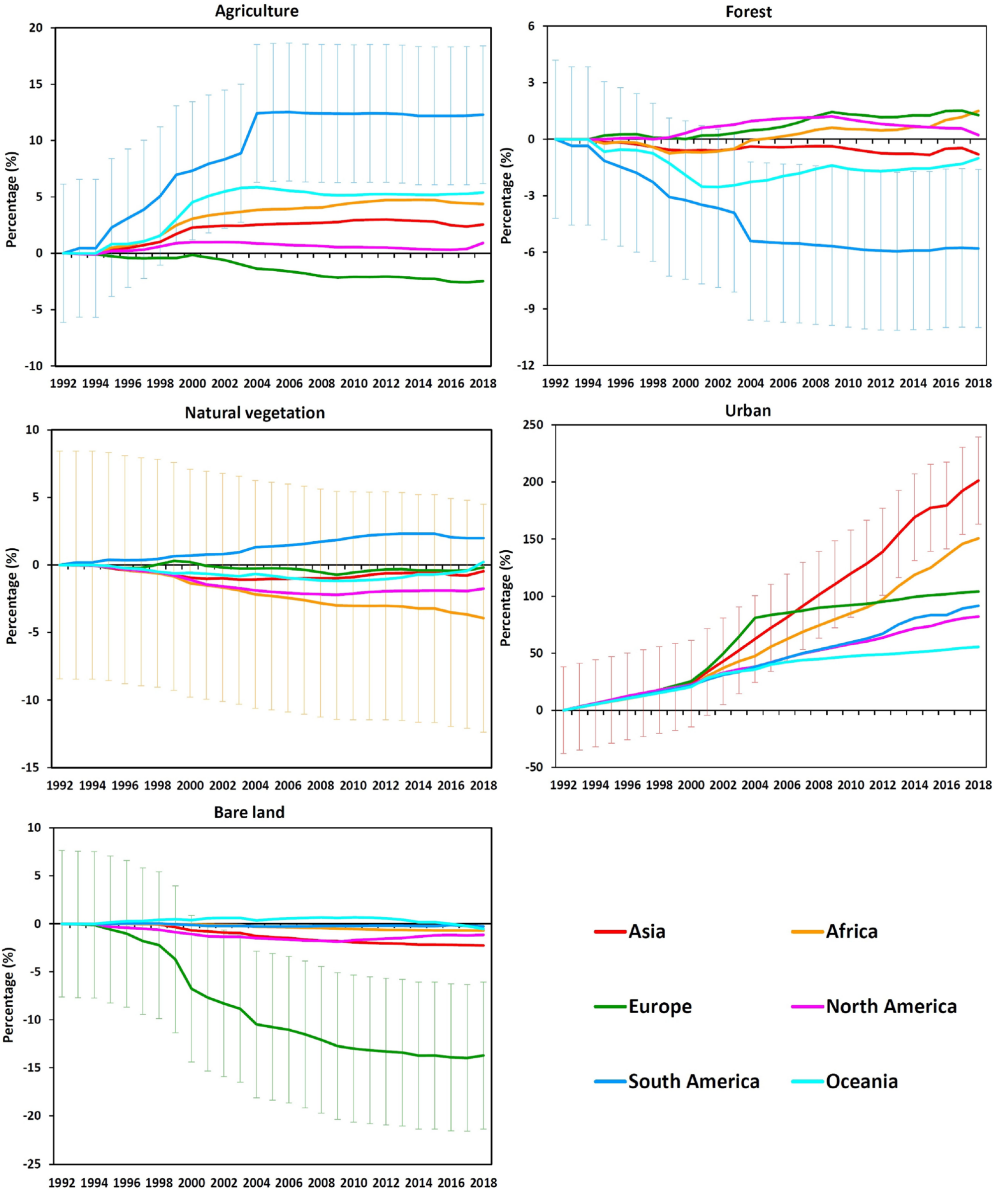
Time-series of the area of each LC type in each continent between 1992 and 2018, expressed as a percentage of the initial area of each LC type. To avoid over-complicating the figure, error bars are provided for the continent showing greatest change in each plot, as an example, representing the margin of error at the 95% confidence interval. Note that in these percentage change plots, for each LC type, the error margin is the same as the example shown, for all other continents.

The trajectories of LC changes within each continent from 1992 to 2018 are shown in Fig. 5, expressed as a percentage (net gain or loss) of the initial area of each LC type in each continent. As suggested by the analysis of the overall net changes above, for most LC types the variability was within the error margins of the LC product. However, changes for some LC types and continents were larger than the error margin. For example, South America exhibited a large increase for agriculture and a large decrease for forest. The trajectories show that these changes were more rapid initially during the study period and stabilised after approximately 2004. There is some evidence that other continents showed similar trajectories to South America for agriculture, but there was more variability between continents in the forest trajectories.

There is evidence of differences in the continental trajectories of natural vegetation, with divergent patterns in South America and Africa, but these were well within the error margins. Urban showed a consistent and substantial increase in all continents over the study period. Growth rates were similar for all continents until around 2000, after which they differed considerably with the highest rates of urban expansion in Asia and lowest in Oceania. Interestingly, Europe showed a more rapid period of urban growth between 2000 and 2004, with much slower growth before and after this period. For bare land, the trajectories for most continents were within the error margins, but for Europe there was a consistent decrease, with some stability towards the end of the time-series, although the absolute net change was relatively small (− 0.049 ± 0.036 million km^2^).

### LC transitions at the global scale

**Figure 6:**
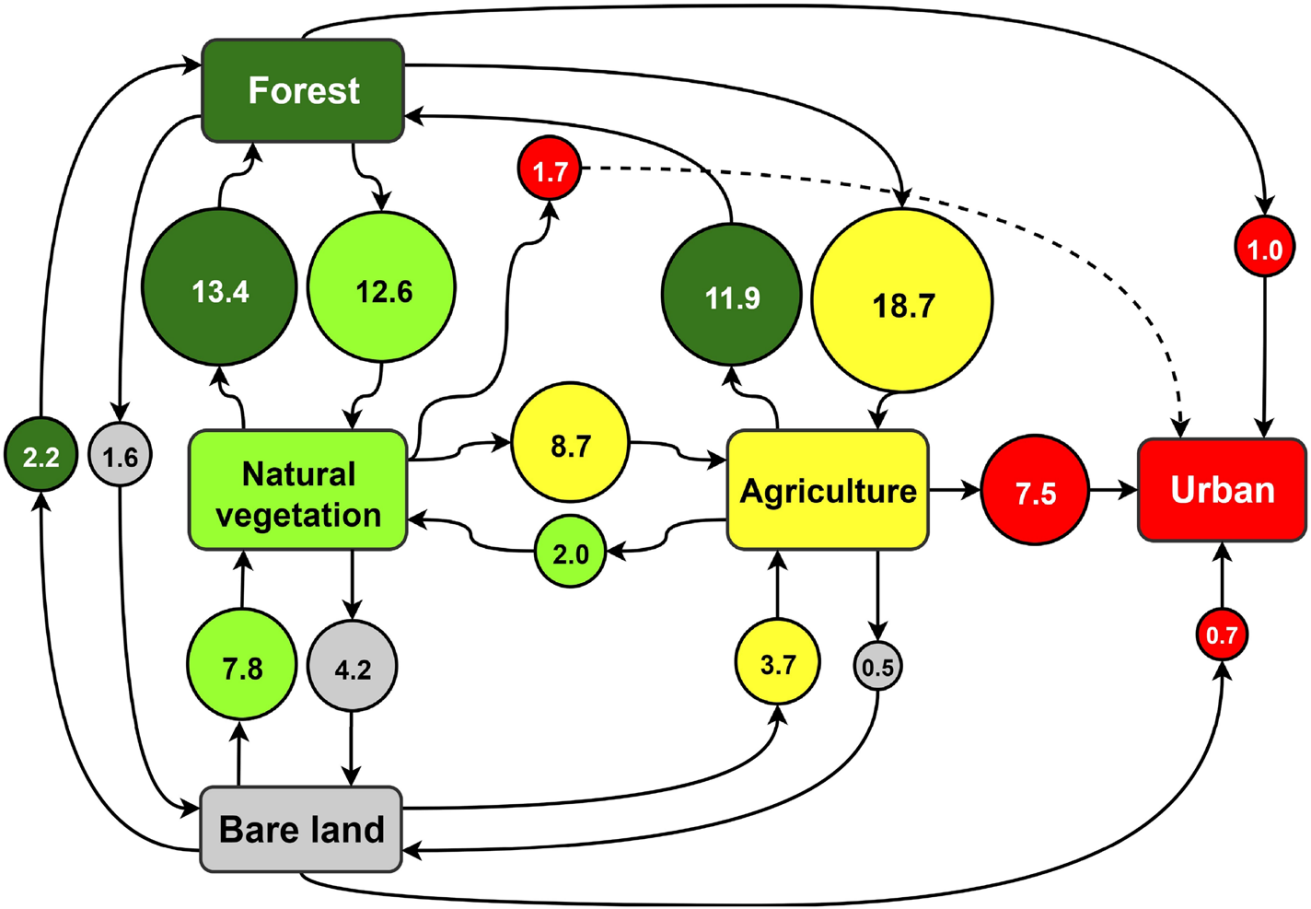
Schematic representation of global LC transitions between 1992 and 2018. The transitions are expressed in percentage terms relative to the total global LC area that changed over this period. Note that the sum of the percentages equals 98.2% as the minor LC transitions involving water bodies were not included. For visualisation purposes, the size of each circle is proportional to the magnitude of the LC transition it represents and exact figures are provided within the circle.

The total area of land that transitioned between different LC types between 1992 and 2018 was 6.8 ± 5.8 million km^2^, equal to 5% of the total ice-free global land area. Figure 6 summarises the transitions that occurred between the five main LC types, where the diameter of the circles represents the area of land that has undergone each transition, expressed in percentage terms relative to the total area of land globally that changed LC type over the study period. The largest transitions were forest cover converting to agriculture followed by natural vegetation converting to forest cover, together contributing 32% of all global transitions. The next largest transitions were forest cover converting to natural vegetation and agriculture converting to forest cover, together contributing 25% of global transitions. This suggests that the major LC dynamics occurred between forest cover, natural vegetation and agricultural land, representing 57% of all global LC transitions between 1992 and 2018. Figure 6 also demonstrates that the transition to urban is unidirectional, as no areas of urban land changed to any other LC class. Hence, we can consider urban development as the endpoint of LC change, which may result from direct conversion from forest, natural vegetation or bare land, or indirectly from these LC types via agriculture. Indeed, the transition from agriculture contributed the greatest amount to urban growth globally (68%).

### LC transitions at the continental scale

The magnitude of transitions between LC types within each continent between 1992 and 2018 are represented schematically in Fig. S2. Asia is the largest continent globally, and so it is not surprising that the largest continental area of LC change was in Asia at 2.36 ± 1.98 million km^2^, equal to 5.3% of the total land area of the continent. The largest transitions were forest cover converting to agriculture and natural vegetation converting to forest cover, accounting for 27% of LC transitions in Asia. The area of transition from bare land to agriculture was the largest among all continents, located mainly in Kazakhstan and Iran. In Asia, agriculture transitioning to urban was the second largest among all continents (after Europe), located mainly in China, Asian Russia and India.

The total area of LC change in South America was 1.1 ± 0.75 million km^2^, equal to 6.2% of the total continental area. The largest transitions were forest converting to agriculture and forest converting to natural vegetation, collectively accounting for 62% of all continental transitions. The areas of transition from forest to agriculture and forest to natural vegetation were the largest among all continents and were located mainly in Brazil, Argentina, Paraguay and Bolivia. Interestingly, the transition from forest cover to agriculture in South America contributed 36% of the corresponding global transition.

The total area of LC change in Europe was 0.65 ± 0.49 million km^2^, equal to 6.5% of the total continental area. The largest transitions were agriculture converting to forest and agriculture converting to urban, collectively accounting for 44% of all continental LC transitions. The areas of transition from agriculture to forest and from agriculture to urban were the largest among all continents, with the latter transition accounting for 79% of urban growth in Europe.

The total area of LC change in Oceania was 0.51 ± 0.50 million km^2^, equal to 6% of the total continental area. The largest transitions included bare land converting to natural vegetation and natural vegetation converting to bare land, collectively accounting for 57% of all continental LC transitions. The areas of transition between natural vegetation and bare land were the largest among all continents, located mainly in Australia. Conversely, the transition from agriculture to urban was responsible for the smallest proportion of urban growth in Oceania (23%), the smallest among all continents.

There were two continents where the overall variability in LC was large but within the error margins associated with ESA-CCE-LC product, therefore we refrain from making definitive statements about these continents. Nevertheless, our results indicate that Africa experienced LC changes covering 1.27 ± 1.50 million km^2^, equal to 4.2% of the total continental land area. The largest transitions were natural vegetation converting to forest and natural vegetation converting to agriculture, collectively accounting for 35% of all LC transitions in Africa. It is noted that the area of transition from natural vegetation to agriculture was the largest among all continents. Furthermore, the total area of LC change in North America was 0.88 ± 0.96 million km^2^, equal to 4% of the total continental area (excluding Greenland). The largest transitions were natural vegetation converting to forest and forest converting to natural vegetation, collectively accounting for 39% of all LC transitions. The area of transition between forest and bare land was the largest among all continents, focused mainly in Canada.

### LC changes at the national scale

**Figure 7:**
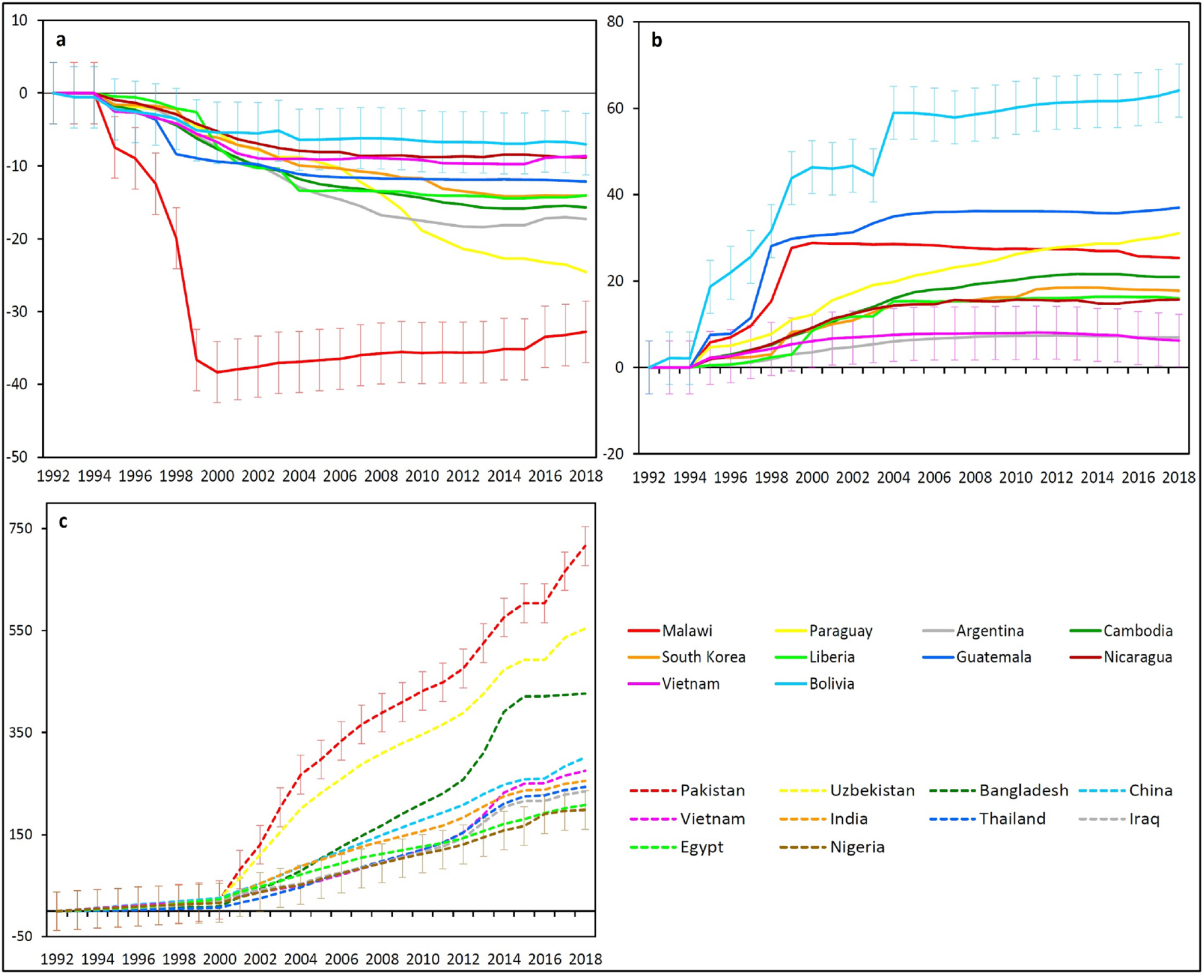
Historical trajectories between 1992 and 2018 for the top 10 global countries in (**a**) forest, (**b**) agriculture and (**c**) urban. Values are expressed as a percentage of the initial area of each LC type. To avoid over-complicating the figure, error bars are provided for the top and bottom lines in each plot, as examples, representing the margin of error at the 95% confidence interval. Note that in these percentage change plots, for each LC type, the error margin is the same for all countries.

**Figure 8:**
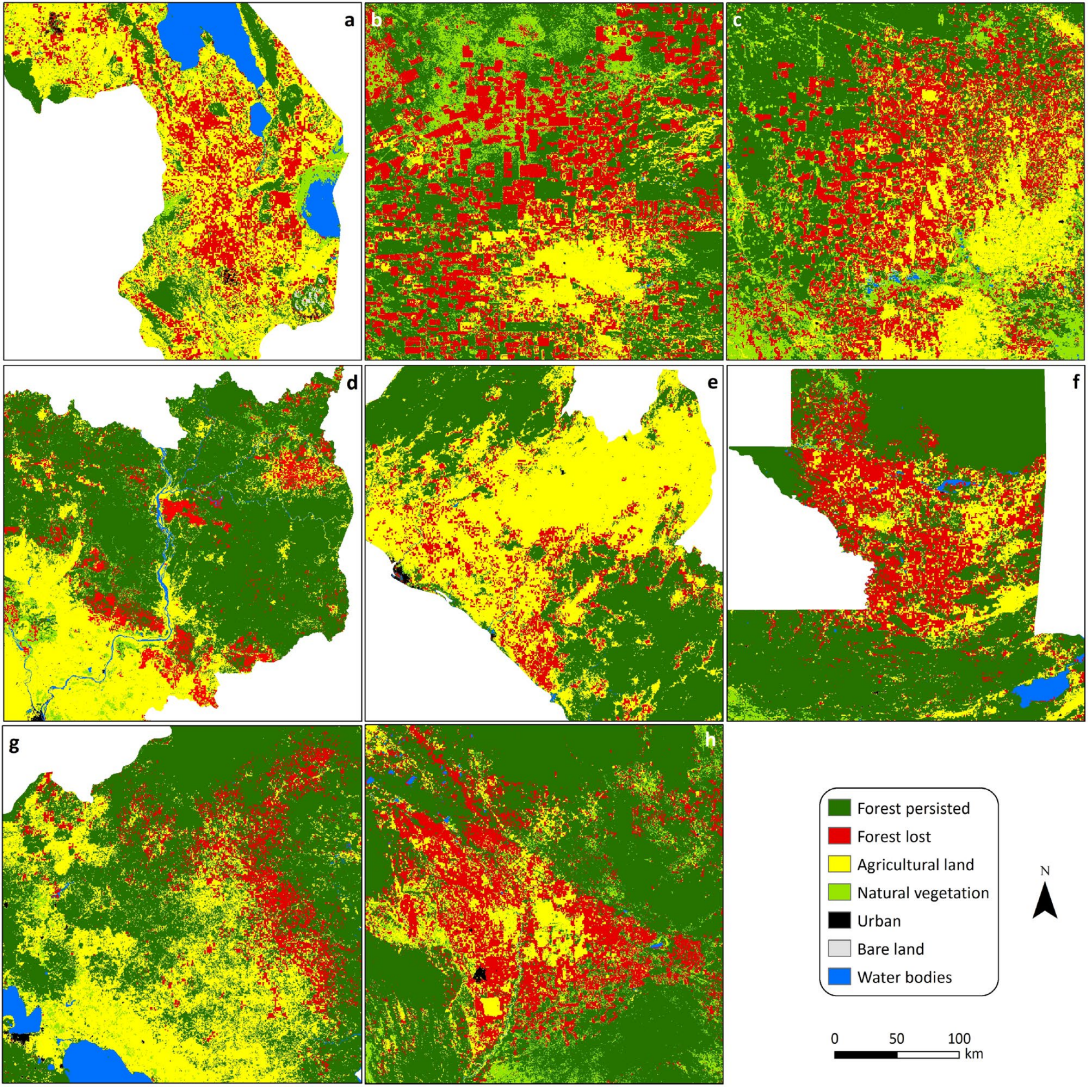
Spatial distribution of forest cover in selected countries with the highest percentages of forest loss between 1992 and 2018: (**a**) Southern Malawi, (**b**) North-western Paraguay, (**c**) Northern Argentina, (**d**) North-eastern Cambodia, (**e**) Central Liberia, (**f**) Northern Guatemala, (**g**) Central Nicaragua, (**h**) Central Bolivia. Note that a consistent map scale has been adopted across all countries. ArcGIS Desktop 10.5^36^ (https://desktop.arcgis.com/en/) was used to generate this figure.

Here, we highlight two of the largest LC changes occurring across the globe (i.e., forest loss and urban expansion), by identifying the 10 countries with the largest percentages of these transitions. Figure 7a shows the historical trajectories in forest cover between 1992 and 2018 for the top 10 countries with the largest percentages of forest loss. It reveals dramatic deforestation in those countries, with losses in forest area ranging from 7% in Bolivia to 33% in Malawi over the study period (Table S2). The amount of forest lost in the top 10 countries was 308,589 ± 103,316 km^2^, accounting for more than 14% of the global total forest loss in 27 years, with Argentina experiencing the largest areal loss of 95,475 ± 24,202 km^2^.The main LC type responsible for these substantial forest cover losses was found to be agricultural land. Consequently, agricultural land experienced substantial gains, ranging from 7% in Argentina to 64% in Bolivia (Fig. 7b). Forest converting to agriculture was responsible for 25% of forest loss in Argentina and up to 85% in Liberia (Table S2). For visualisation purposes, Fig. 8 shows eight of the selected countries in more detail, highlighting the substantial areas of forest cover loss in those countries.

**Figure 9:**
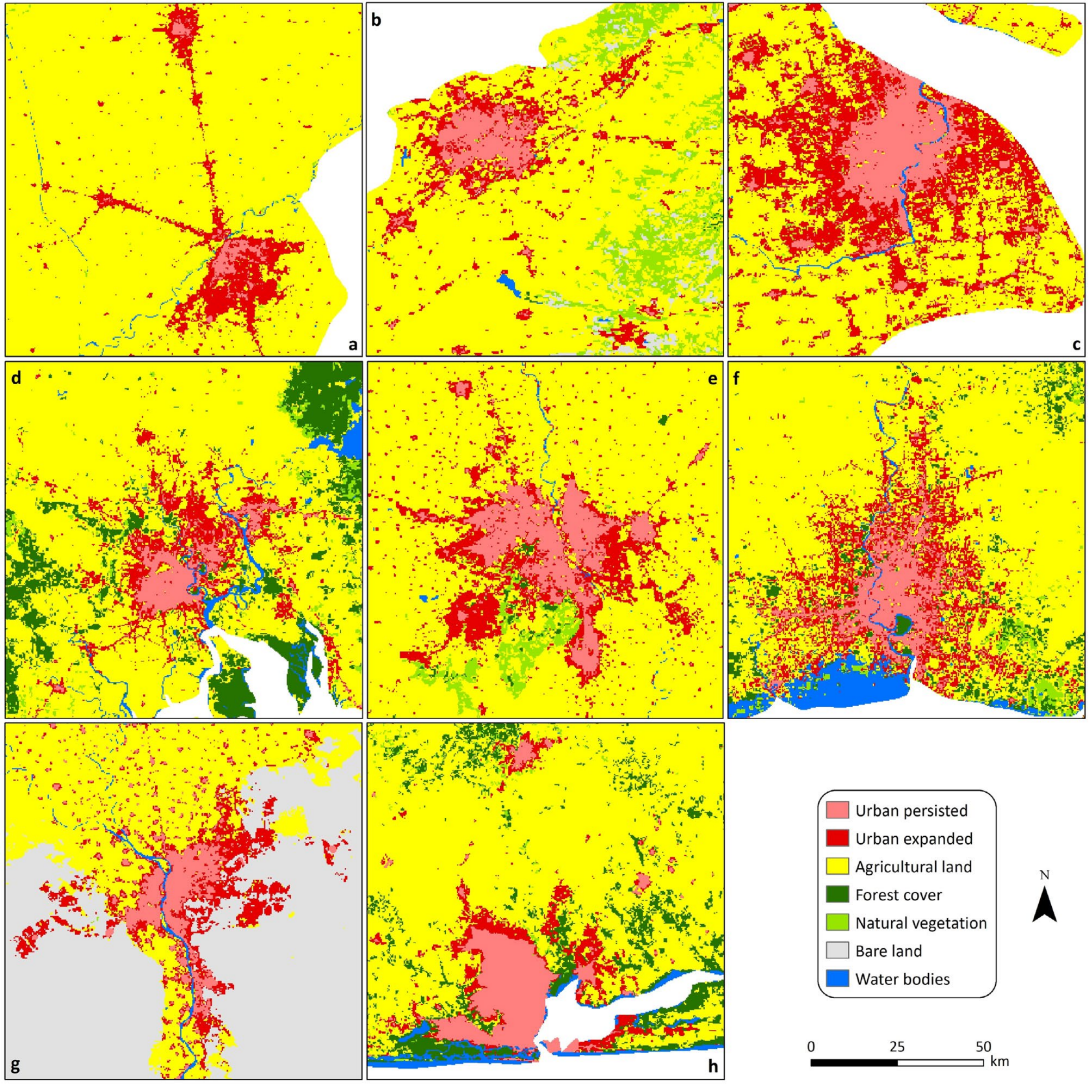
Spatial distribution of urban land in selected major urban cities within eight of the 10 countries with the largest percentages of urban expansion between 1992 and 2018: (**a**) Lahore, Pakistan (**b**) Tashkent, Uzbekistan (**c**) Shanghai, China (**d**) Ho Chi Minh, Vietnam (**e**) New Delhi, India (**f**) Bangkok, Thailand (**g**) Greater Cairo, Egypt (**h**) Lagos, Nigeria. Note that a consistent map scale has been adopted across all countries. ArcGIS Desktop 10.5^36^ (https://desktop.arcgis.com/en/) was used to generate this figure.

Figure 7c shows the historical trajectories in urban land from 1992 to 2018 within the 10 countries with the largest percentages of urban expansion. It shows the widespread and rapid increases in urban area, with changes over the study period ranging from 199% in Nigeria to 716% in Pakistan (Table S3). The amount of urban land gained in these 10 countries was about 250,968 ± 76,038 km^2^, accounting for about 25% of the global urban expansion in 27 years, with China experiencing the largest urban area gained, at 175,802 ± 52,823 km^2^.The main LC type lost because of this substantial urban expansion was agriculture. The transition from agriculture to urban accounted for an average of 82% of urban gain and 56% of agriculture loss for the 10 countries (Table S3). This demonstrates the historical and ongoing threats of urban expansion on neighbouring productive agricultural land. For visualisation purposes, Fig. 9 shows selected major urban cities within eight of the top 10 countries, highlighting the dramatic urbanisation in those countries.

## Discussion and conclusions

The findings of this study provide new insights into the characteristics of LC dynamics across the globe at multiple spatial scales over an extended period of time. To the best of our knowledge, this study is the first to provide a comprehensive analysis of all LC changes across global, continental and national scales between 1992 and 2018 by quantifying LC gains and losses, trajectories and transitions using the ESA-CCI-LC annual time-series at 300 m spatial resolution, and considers the uncertainty in the LC dataset.

While several studies have been undertaken recently to quantify LC changes at the global scale using the ESA-CCI-LC dataset, these have considered global changes from different perspectives including investigating plant functional types^31^ (PFTs) and landscape ecology^28^. Huang et al.^37^ analysed the global urban expansion and its associated implications on the Net Primary Productivity (NPP) of cropland. van Vliet^33^ analysed the consequences of global urban expansion on the direct and indirect changes in LC, particularly the neighbouring cropland. Mousivand and Arsanjani^32^ quantified LC changes at the global scale using this dataset. However, they did not consider continental or national-scale LC changes or the magnitude of transitions between different combinations of LC types, and their findings were based on an analysis up to 2015. Four other studies focused on quantifying related forest cover changes in Southeast Asia^29,35^, developing countries^30^ and China^34^. Very few studies have been carried out on a global scale using fine-resolution imagery. Song et al.^38^ developed an annual fine-resolution global product consisting of three land cover types, namely, tree canopy cover, short vegetation cover and bare ground cover using satellite sensor observations over the period 1982 to 2016. Jokar Arsanjani^39^ characterised global LC changes in 2000 and 2010 and reported large changes across all continents using a relatively fine resolution dataset (GlobeLand30) based on the archive of Landsat imagery.

The comprehensive results produced here using the ESA-CCI-LC dataset are comparable with the findings of other more specific studies that have used fine-resolution data for the analysis of trajectories in, and mapping of cropland^40^, forest cover change^41^ and urbanisation^42^. This study, thus, demonstrates that the release of the ESA-CCI-LC global dataset has enabled a step-change in understanding global LC changes that have occurred over a period of more than a quarter of a century, without significant spatial resolution trade-off^16^. Our results also show that the ESA-CCI-LC data can provide valuable insights into LC trajectories, transitions and the locations of changes at continental and national scales, as well as at the global scale^33^. This is enabled by the high temporal consistency of the ESA-CCI-LC product.

Like any remote sensing based dataset, there are some associated limitations^43^ with the ESA-CCI dataset. For example, different sources of input data were used to generate the product, notably the AVHRR sensor from 1992 to 1999 and SPOT-VGT and MERIS from 1999 and 2003 onwards, respectively. The coarser spatial resolution of the AVHRR data was effectively resampled to 300 m to generate the final LC product, but the original 1 km resolution of the data may impose limitations with the data from the earlier years of the LC time series. Likewise, the change in the sources of input data may account for some of the observed changes in LC, such as the notable differences in urban extent after the year 2000. The confusion matrix (Table S4), which quantifies the correspondence between the ESA-CCI LC data and independently determined LC reference samples, indicates that the accuracy of the individual LC classes is generally high and typical of a remotely sensed product, although the urban class does have a low producer’s accuracy. This meant that when the errors revealed in the confusion matrix were used as the basis to correct the estimated areas of the LC classes, the largest correction was applied to the urban class. While the correction method we used^44^ is well established and logical, it can place a strong emphasis on the specific reference and LC map data used in the accuracy assessment. Furthermore, the accuracy assessment used in this study was derived from the LC map of year 2015 and the confusion matrix derived for that year was used to correct the LC data for all other years. While the accuracy assessment was rigorous and used a large reference dataset, it may be preferable to collect reference data from several other years and derive accuracy assessments from those years of LC data.

Nevertheless, using the confusion matrix (Table S4) as part of the correction method^44^, it was possible to estimate the margin of error at the 95% confidence interval for the LC changes observed. This demonstrated that at the global scale the observed changes for most classes (other than urban) were smaller than the margin of error. This is because at the global scale, despite the absolute areas of change being large (e.g. 0.90 million km^2^ for agriculture) these only represented a small percentage of the total global extent of each LC class (e.g. 3% change for agriculture) which is smaller than the margin of error (e.g. ± 7.4% for the forest to agriculture transition). As we consider smaller spatial extents then LC changes can make up a much larger percentage of the total area of each LC class. If, for example, we considered an area where all of the forest was converted to agriculture, the change would be 100% while the margin of error would remain at ± 7.4%. Hence, as we can see from Tables S1, as we move from global to continental scales, for many LC classes the observed changes become comparable with the margin of error. Furthermore, as we move to the scale of individual countries, the LC changes considerably exceed the margin of error.

While several recent studies have reported the results of their analyses of global LC changes using remotely sensed LC products, many have not considered the errors associated with such products and the consequent impact on the confidence of the change results. Our findings demonstrate the importance of accounting for errors in the LC product^45^. For example, the observed global net change in forest was less than the margin of error of the LC product (which is similar to other LC products, as noted above). Therefore, there remains some uncertainty in determining the direction (positive or negative) of the global net change in forest. This may explain the apparently contradictory findings from the Global Forest Resources Assessment 2015^46^ which reported a global net loss in forest of 1.29 million km^2^ from 1990 to 2015, and those of Song et al.^38^, who reported a global net gain in forest of 2.24 million km^2^ from 1982 to 2016. To some extent, our findings are in line with those of Hansen et al^41^ who reported a global gross forest loss of 2.3 million km^2^ between 2000 and 2012.

The observed changes for most other LC types were within the margins of error at global and continental scales as determined from an accuracy assessment of the LC product. However, such an accuracy assessment evaluates the correspondence between the LC product and reference data and uncertainty in either can lead to lower accuracy^47^. A multitude of factors result in error associated with LC reference data collected via techniques such as field surveys or manual interpretation of fine-resolution imagery^48^, as in this study. Hence an accuracy assessment using such reference data can be considered as a measure of the correspondence between two different techniques, rather than a measure of the disparity between a LC product and the ‘truth’. Moreover, such an accuracy assessment does not assess the internal consistency of the remote sensing dataset itself, which in many cases, due to the high geometric and measurement precision of the sensing device, is expected to be greater than that of the reference data. In this sense, the accuracy assessment may be considered as a conservative measure of the value of the ESA-CCI-LC product^49^, but does provide some context with which to interpret the LC changes observed.

Our analysis of the global, fine-resolution LC dataset allowed identification of when and where changes have taken place, allowing us to focus on hotspots where changes can be observed irrespective of the measurement uncertainty. The maps produced from this analysis provide information on the global distribution of LC changes, at fine spatial resolution, and identify locations with high dynamism. These include countries with the highest levels of forest loss and urban growth, for which a more in-depth analysis was undertaken. Such findings provide valuable quantitative insights into the recognised contributions of agricultural expansion to forest cover loss^33^, particularly via the extensification of cultivation practices in South America^50^ and Southeast Asia^29^. Further consideration of the drivers and potential impacts of LC changes within individual continents and countries is provided in Supplementary Information S1.

The world now faces several environmental sustainability challenges, most of which are considered the consequences of recent LC change^11^, and our results both highlight and quantify the magnitude of these changes. Our results show that the global increase in urban areas between 1992 and 2018 was equivalent to the size of Egypt. Urban was the only LC type that experienced consistent annual gain. This is to be expected since urbanisation is generally considered to be the end-point of a one-way process and, hence, it is very unlikely that it will convert to any other LC type once established^51^. To some extent, our findings are in line with those of van Vliet^33^ who reported a net gain in urban area of 0.38 million km^2^ from 1992 to 2015, and Gong et al.^42^ who reported an increase of 0.48 million km^2^ from 1990 to 2018. Urban areas are expected to continue expanding over the coming decades with consequent environmental impacts^52^.

The sustainable development goals (SDGs) of the United Nations Development Programme (UNDP) were introduced in 2015 as a global incentive towards maintaining the sustainability of the Earth’s resources and providing better and healthier lives for hundreds of millions of people^53^. SDG 2 is aimed at ending hunger, achieving food security and promoting sustainable agriculture^53^. However, it has been recognised that achieving food security for a rapidly growing global population may be hampered due to restrictions in the amount of available arable land^51^. The findings of the present study demonstrate the gravity of this situation and a key issue is the loss of arable land to urban expansion^54^. our findings further emphasise this problem as the global increase in urban areas by 125% was largely at the expense of agricultural land, a total loss equivalent to the area of Ecuador. It has been recognised that fulfilling the increasing global demand for food has come at the expense of natural resources, for example, via natural habitat and biodiversity loss^52^. Our findings confirm the magnitude of these effects, as the expansion of agricultural land has been the major contributor to the loss of natural vegetation and forest across all continents. This highlights the pressing need for alternative solutions to food security such as agricultural intensification^55^ and converting bare land to agricultural land^4^, although the latter may itself come at substantial financial costs.

Policy to deliver environmentally sustainable routes to food security must be based on a solid evidence base, with information on global LC changes as a fundamental component, as provided here. Moreover, deep understanding of the human–environment interaction system and, in particular, the magnitude of recent LC changes and the factors driving these changes, is required to address the grand sustainability challenges facing humanity, not least the SDGs of the UNDP. The analysis produced here on the LC changes that have occurred over the last quarter of a century provides crucial information in support of these goals.

## Methods

### The ESA-CCI-LC dataset

The ESA recently launched its CCI-LC programme aiming to generate high-quality satellite-derived LC data at the global level. The ESA-CCI-LC dataset provides temporally consistent global LC coverage at a spatial resolution of 300 m on an annual basis from 1992 to 2018. The ESA-CCI-LC dataset consists of a raster (pixel) based map for each year. This is the longest period of annual LC data available to-date, and it is considered the first consistent time-series of global LC coverage at relatively fine spatial resolution. Unlike other global LC datasets generated based on a single-year or a single-sensor basis, this dataset is derived using multiple Earth observation sensors, including MERIS, SPOT-VEGETATION, PROBA-V and AVHRR. The ESA-CCI-LC dataset assigns the LC into 37 classes (22 global classes and 15 regional classes) based on a classification scheme developed by the Food and Agriculture Organization^43^ (FAO).

Being a raster-based product the minimum mappable unit (MMU) of the ESA-CCI-LC data is effectively equal to the spatial resolution of the data, at 300 × 300 m. It has been argued that in raster-based remotely sensed imagery the smallest observable feature that can be identified reliably is four contiguous pixels in size (i.e. 600 × 600 m). Nevertheless, because the analysis in this paper was based on pixel-by-pixel differences (and not on objects), the MMU of 300 × 300 m is considered valid and the ESA-CCI-LC dataset is appropriate for the objectives of the study.

### Reclassification of the ESA-CCI-LC dataset

**Figure 10:**
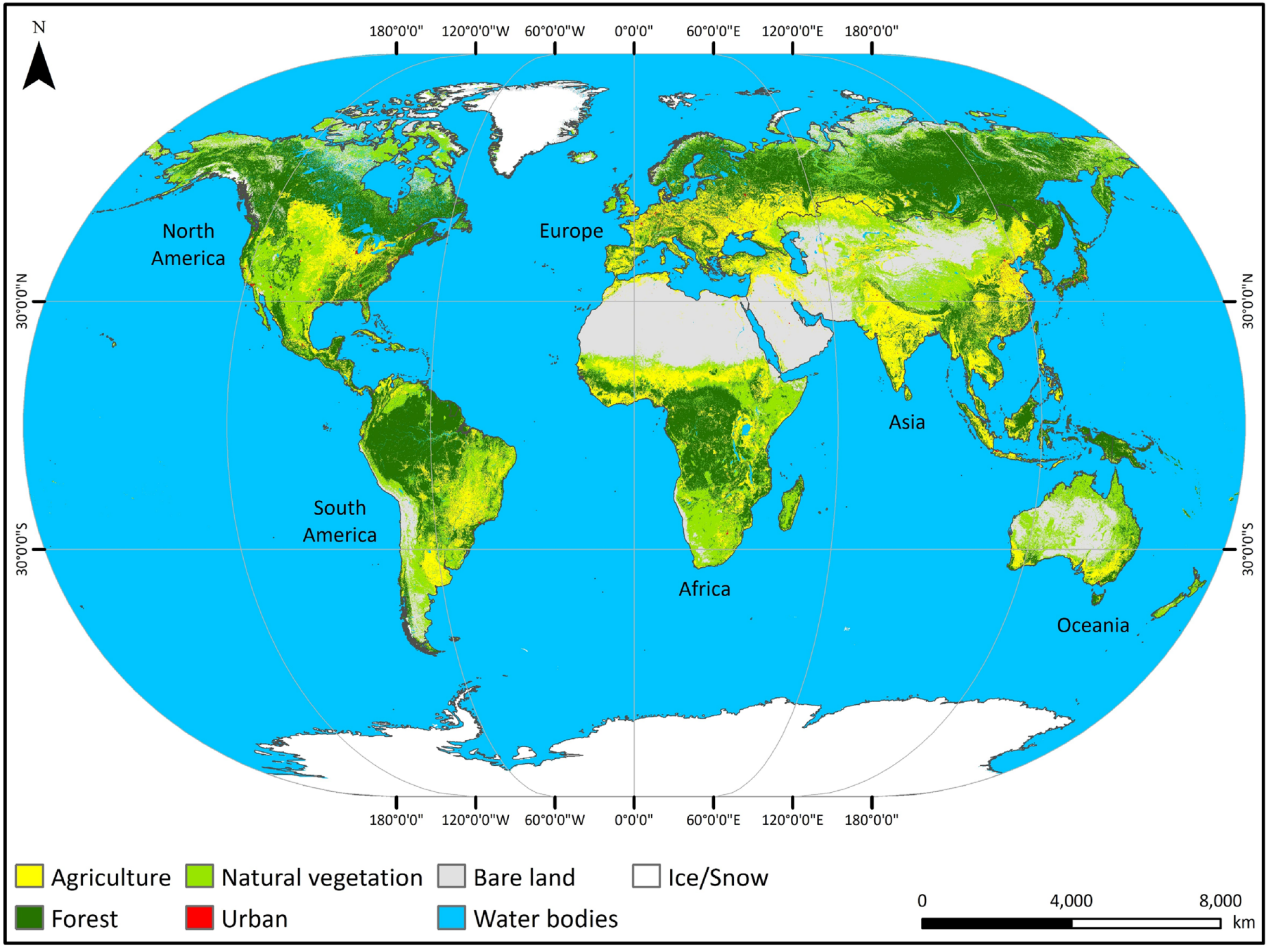
Global distribution of LC types in 2018. ArcGIS Desktop 10.5^36^ (https://desktop.arcgis.com/en/) was used to generate this figure.

To provide clarity in the analysis of LC changes, seven main LC types, namely: agriculture, forest, natural vegetation, urban, bare land, water bodies and ice/snow were generated by combining the relevant classes in the original ESA-CCI-LC data, via a reclassification (Table S5). Global data of the seven LC types were generated through the reclassification process for each of the years from 1992 to 2018 and used in the subsequent analysis. For illustrative purposes, Fig. 10 shows the global distribution of the main LC types in 2018.

### Quantifying LC changes

The seven class LC data were analysed using the procedures described below at three scales: by using the entire global dataset, by extracting data relating to each individual continent, and by using data for selected individual countries that experienced the largest changes of particular LC classes. To quantify the overall LC gains and losses, a map was extracted from the reclassified ESA-CCI-LC data for 1992 and 2018 for each of the five LC types. Then a difference map (between 1992 and 2018) was generated showing areas gained and lost for each LC type. The total areas of gross gains and losses were calculated by multiplying the total number of pixels representing a gain or a loss by the pixel area, for each LC type. Net changes were quantified by calculating the difference between gross gain and gross loss, for each LC type. Furthermore, to map the spatial distribution of the LC gains and losses, the generated difference maps were used. A spatial aggregation technique was used for visualising the data appropriately at the global scale, and this was based on a 10 × 10 pixel aggregation, thereby creating global maps of gains and losses for each LC type at a 3 km spatial resolution.

To quantify the LC trajectories from 1992 to 2018, the total area of each LC type in each year was computed, and this was calculated by multiplying the total pixel count by the pixel area. Finally, the area of land involved in transitions between all combinations of LC types from 1992 to 2018 was quantified. This was achieved by generating difference maps for each LC type, showing the areas that had transitioned from that LC type in 1992 to each of the other four LC types in 2018. This has produced maps representing the areas involved in each of the possible transition types. Then the total area for each transition type was computed by multiplying the total pixel count for that transition type by the pixel area. To represent LC transitions in our schematic model, the area of land involved in each transition type was expressed in percentage terms relative to the total area of LC change between 1992 and 2018.

### Accuracy assessment and area correction

An accuracy assessment of the ESA-CCI-LC product^43^ used a sample of 1499 locations across the globe to quantify the correspondence between the LC class allocated in the ESA-CCI-LC product for that location and the LC class as determined from an independent validation dataset. We used the data generated from the accuracy assessment, based on the original 22 global ESA-CCI-LC classes, and combined the data for relevant groups of classes (as in Table S5) to produce a confusion matrix for the seven aggregated classes that were used in this study. A confusion matrix is able to quantify the thematic errors of commission (where pixels are assigned to a particular LC class at locations where there is a different LC in the reference data) and omission (where pixels are not assigned to a particular LC class, but their locations have that LC in the reference data). Using an established method^44^, the errors quantified in the confusion matrix were used to correct the mapped areas (i.e., derived from pixel counts) of each class and express the uncertainty of the estimated area as a margin of error at the 95% confidence interval. The uncertainty in LC change was expressed as the summation in quadrature of the margins of error of the maps of both LC classes that were used to quantify the change. The confusion matrix for the seven classes expressed as the corrected area of each class as a proportion of total area is shown in Table S4, along with accuracy metrics. As explained in the discussion section, this uncertainty analysis can be considered conservative.

## Supplementary Information

Supplementary Information.
