# Supplementary material for: Global land cover trajectories and transitions
Source: Sci Rep. 2021 Jun 17;11:12814. doi: 10.1038/s41598-021-92256-2 (PMC8211844; doi:10.1038/s41598-021-92256-2)
Supplement: Supplementary file 1 — Supplementary Information. [file 41598_2021_92256_MOESM1_ESM.docx]

**Global land cover trajectories and transitions**

Taher M. Radwan ^1,2,*^, G. Alan Blackburn ^1^, J. Duncan Whyatt ^1^ and Peter M. Atkinson ^1,3,4^

1. Lancaster Environment Centre, Lancaster University, Lancaster LA1 4YQ, UK; [t.radwan@lancaster.ac.uk](mailto:t.radwan@lancaster.ac.uk); [taher.radwan@alexu.edu.eg](mailto:taher.radwan@alexu.edu.eg) (T.M.R); alan.blackburn@lancaster.ac.uk (G.A.B.); d.whyatt@lancaster.ac.uk (J.D.W); pma@lancaster.ac.uk (P.M.A.)

2. Department of Soil and Water Sciences, Faculty of Agriculture (El-Shatby), Alexandria University, Alexandria 21545, Egypt

3. Institute of Geographic Sciences and Natural Resources Research, Chinese Academy of Sciences, Beijing 100101, China

4. Geography and Environmental Science, University of Southampton, Highfield, Southampton SO17 1BJ, UK

* Correspondence: Taher M. Radwan

Email: [t.radwan@lancaster.ac.uk](mailto:t.radwan@lancaster.ac.uk); [taher.radwan@alexu.edu.eg](mailto:taher.radwan@alexu.edu.eg)

**Supplementary Information**

**
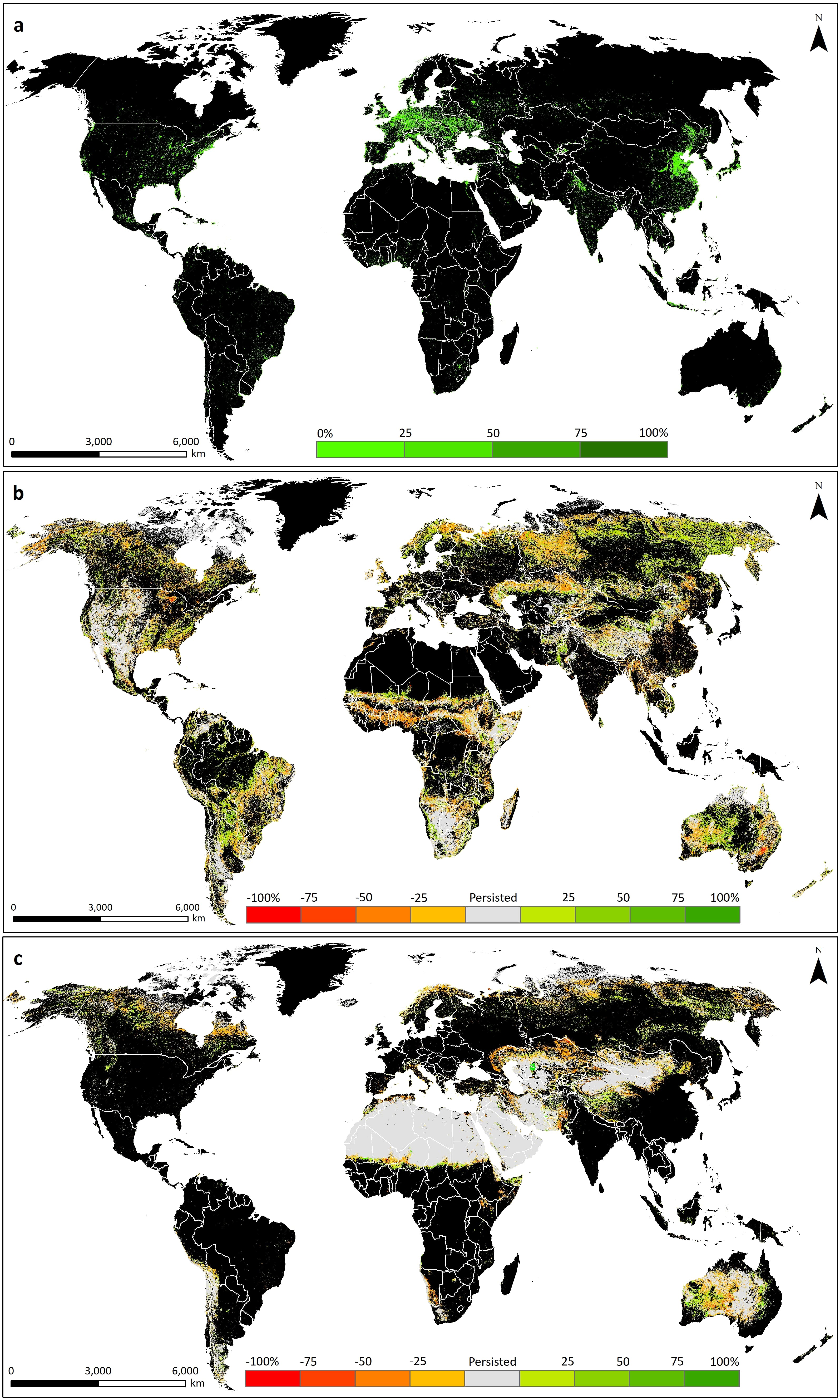
**

**Fig. S1: Spatial distribution of LC change** **between 1992 and 2018**. (**a**) urban, (**b**) natural vegetation, and (**c**) bare land. The original data were aggregated to a 3 km spatial resolution for visualisation. Black areas are terrestrial zones where the LC type was absent in both 1992 and 2018. ArcGIS Desktop 10.5^36^ (<https://desktop.arcgis.com/en/>) was used to generate this map.

**
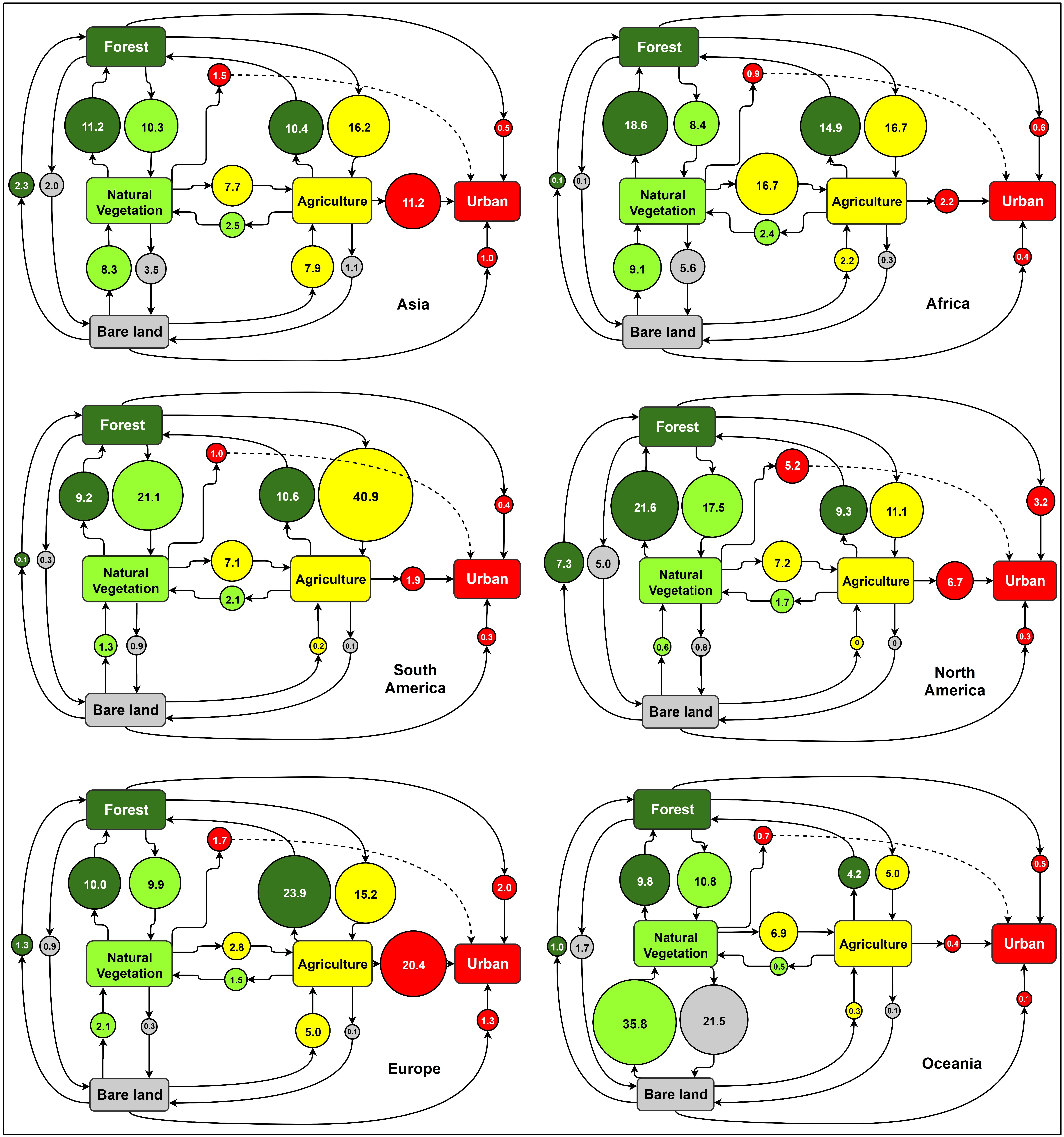
**

**Fig. S2: Schematic representation of continental LC transitions between 1992 and 2018.** The transitions are expressed in percentage terms relative to the total LC area in each continent that changed over this period. For visualisation purposes, the size of each circle is proportional to the magnitude of the LC transition it represents and exact figures are provided within the circle.

**Table S1:** Changes in the main five LC classes between 1992 and 2018 at global and continental scales. Areas are expressed in million km^2^ (the minus sign means a loss). The margin of error at the 95% confidence interval is shown for LC changes and transitions.

| **LC class** | **Agriculture** | **Forest** | **Natural vegetation** | **Urban** | **Bare land** |
| --- | --- | --- | --- | --- | --- |
| Global | 0.905 ± 2.510 | -0.414 ± 2.364 | -0.283 ± 3.437 | 1.019 ± 0.777 | -0.479 ± 3.09 |
| Asia | 0.286 ± 0.984 | -0.102 ± 0.752 | -0.035 ± 0.913 | 0.462 ± 0.277 | -0.258 ± 1.221 |
| Africa | 0.233 ± 0.472 | 0.101 ± 0.406 | -0.259 ± 0.769 | 0.072 ± 0.049 | -0.073 ± 1.125 |
| North America | 0.031 ± 0.295 | 0.017 ± 0.467 | -0.110 ± 0.739 | 0.192 ± 0.184 | -0.022 ± 0.204 |
| South America | 0.422 ± 0.316 | -0.462 ± 0.459 | 0.079 ± 0.483 | 0.056 ± 0.050 | -0.003 ± 0.129 |
| Europe | -0.107 ± 0.372 | 0.045 ± 0.205 | -0.002 ± 0.157 | 0.226 ± 0.187 | -0.049 ± 0.036 |
| Oceania | 0.040 ± 0.066 | -0.012 ± 0.073 | 0.044 ± 0.354 | 0.013 ± 0.016 | -0.073 ± 0.345 |

**Table S2:** Top 10 countries with the highest percentages of forest loss and the impact on neighbouring agricultural land. The margin of error at the 95% confidence interval is shown for LC changes and transitions.

| Country | Forest loss (%) | Forest loss (km^2^) | Forest to agriculture (km^2^) | Forest to agriculture (%) of all transitions | Forest to agriculture (%) of total forest loss | Forest to agriculture (%) of total agric. gain |
| --- | --- | --- | --- | --- | --- | --- |
| Malawi | 32.8 | 16,502 ±1,982 | 15,196 ±1,129 | 74.5 | 92.1 | 98.6 |
| Paraguay | 24.6 | 62,015 ±11,997 | 24,184 ±1,796 | 32.9 | 39.0 | 91.6 |
| Argentina | 17.3 | 95,475 ±24,202 | 37,539 ±2,788 | 24.9 | 39.3 | 70.2 |
| Cambodia | 15.7 | 16,250 ±5,450 | 11,791 ±867 | 56.5 | 72.6 | 78.2 |
| South Korea | 14.1 | 9,264 ±3,016 | 8,671 ±644 | 62.7 | 93.6 | 99.0 |
| Liberia | 14.0 | 8,513 ±2,797 | 8,423 ±626 | 85.2 | 98.9 | 99.9 |
| Guatemala | 12.1 | 13,398 ±4,464 | 12,326 ±916 | 68.4 | 92.0 | 99.7 |
| Nicaragua | 8.9 | 9,146 ±4,390 | 8,193 ±609 | 70.5 | 89.6 | 99.7 |
| Vietnam | 8.7 | 16,451 ±7,309 | 10,603 ±788 | 33.3 | 64.5 | 68.9 |
| Bolivia | 7.0 | 61,575 ±37,709 | 36,840 ±2,736 | 42.8 | 59.8 | 96.4 |

**Table S3:** Top 10 countries with the highest percentages of urban expansion and the impact on neighbouring agricultural land. The margin of error at the 95% confidence interval is shown for LC changes and transitions.

| Country | Urban expansion (%) | Urban expansion (km^2^) | Agriculture to urban (km^2^) | Agriculture to urban (%) of all transitions | Agriculture to urban (%) of total urban gain | Agriculture to urban (%) of total agric. loss |
| --- | --- | --- | --- | --- | --- | --- |
| Pakistan | 715.8 | 6,968 ±1,742 | 6,243 ±2,407 | 11.2 | 89.6 | 65.7 |
| Uzbekistan | 553.7 | 6,185 ±1,610 | 5,432 ±2,095 | 23.6 | 87.8 | 61.5 |
| Bangladesh | 426.2 | 1,535 ±418 | 1,460 ±563 | 24.7 | 95.1 | 45.3 |
| China | 301.2 | 175,802 ±52,823 | 142,584 ±54,984 | 23.2 | 81.1 | 61.4 |
| Vietnam | 275.1 | 4,457 ±1,365 | 4,254 ±1,640 | 13.4 | 95.5 | 54.4 |
| India | 255.4 | 35,565 ±11,192 | 32,257 ±12,439 | 27.9 | 90.7 | 53.7 |
| Thailand | 243.6 | 5,280 ±1,684 | 5,054 ±1,949 | 20.0 | 95.7 | 50.1 |
| Iraq | 234.7 | 3,062 ±1,008 | 1,324 ±511 | 15.2 | 43.2 | 44.0 |
| Egypt | 208.2 | 3,860 ±1,318 | 2,617 ±1,009 | 30.6 | 67.8 | 91.0 |
| Nigeria | 198.7 | 8,254 ±2,878 | 6,204 ±2,392 | 6.6 | 75.2 | 32.9 |

**Table S4:** Confusion matrix for the seven aggregated LC classes used in this study with cell entries expressed as the estimated proportion of area, with user’s and producer’s accuracies of the individual classes and the overall accuracy of the LC dataset. Mapped classes are the rows and reference classes are the columns.

| LC class | Agriculture | Forest | Natural vegetation | Urban | Bare land | Water bodies | Ice/snow | Total | User’s | Producer’s | Overall |
| --- | --- | --- | --- | --- | --- | --- | --- | --- | --- | --- | --- |
| Agriculture | 0.1527 | 0.0063 | 0.0117 | 0.0029 | 0.0015 | 0.0000 | 0.0000 | 0.1751 | 0.87 | 0.77 | 0.82 |
| Forest | 0.0094 | 0.2503 | 0.0310 | 0.0006 | 0.0033 | 0.0017 | 0.0006 | 0.2968 | 0.84 | 0.93 |  |
| Natural vegetation | 0.0325 | 0.0103 | 0.1351 | 0.0022 | 0.0229 | 0.0015 | 0.0022 | 0.2067 | 0.65 | 0.70 |  |
| Urban | 0.0007 | 0.0000 | 0.0000 | 0.0043 | 0.0000 | 0.0000 | 0.0000 | 0.0050 | 0.86 | 0.36 |  |
| Bare land | 0.0039 | 0.0010 | 0.0157 | 0.0020 | 0.1618 | 0.0010 | 0.0059 | 0.1912 | 0.85 | 0.84 |  |
| Water | 0.0003 | 0.0000 | 0.0006 | 0.0000 | 0.0000 | 0.0247 | 0.0000 | 0.0256 | 0.96 | 0.86 |  |
| Ice/snow | 0.0000 | 0.0000 | 0.0000 | 0.0000 | 0.0038 | 0.0000 | 0.0957 | 0.0995 | 0.96 | 0.92 |  |
| Total | 0.1995 | 0.2679 | 0.1941 | 0.0120 | 0.1933 | 0.0288 | 0.1044 | 1.0000 |  |  |  |

**Table S5:** The LC types analysed in this research and the original ESA-CCI-LC classes from which they were derived (ESA-CCI-LC: Product user guide: Version 2, available at <http://maps.elie.ucl.ac.be/CCI/viewer/download/ESACCI-LC-Ph2-PUGv2_2.0.pdf>).

| LC types used in this study | Original LC class codes in the ESA-CCI-LC dataset combined to form the LC types used in this study | Description of the original LC classes in the ESA-CCI-LC dataset |
| --- | --- | --- |
| 1. Agriculture | 10, 11, 12 | Rainfed cropland |
|  | 20 | Irrigated cropland |
|  | 30 | Mosaic cropland (> 50%)/natural vegetation (tree, shrub, herbaceous cover) (< 50%) |
|  | 40 | Mosaic natural vegetation (tree, shrub, herbaceous cover) (> 50%)/cropland (< 50%) |
| 1. Forest | 50 | Tree cover, broadleaved, evergreen, closed to open (> 15%) |
|  | 60, 61, 62 | Tree cover, broadleaved, deciduous, closed to open (> 15%) |
|  | 70, 71, 72 | Tree cover, needleleaved, evergreen, closed to open (> 15%) |
|  | 80, 81, 82 | Tree cover, needleleaved, deciduous, closed to open (> 15%) |
|  | 90 | Tree cover, mixed leaf type (broadleaved and needleleaved) |
|  | 100 | Mosaic tree and shrub (> 50%)/herbaceous cover (< 50%) |
|  | 160 | Tree cover, flooded, fresh or brakish water |
|  | 170 | Tree cover, flooded, saline water |
| 1. Natural vegetation | 110 | Mosaic herbaceous cover (> 50%)/tree and shrub (< 50%) |
|  | 120, 121, 122 | Shrubland |
|  | 130 | Grassland |
|  | 140 | Lichens and mosses |
|  | 180 | Shrub or herbaceous cover, flooded, fresh-saline or brakish water |
| 1. Urban | 190 | Urban |
| 1. Bare land | 150, 152, 153, 200, 201, 202 | Bare areas and sparse vegetation |
| 1. Water bodies | 210 | Water |
| 1. Ice/Snow | 220 | Permanent ice and snow |

**S1: Drivers and implications of LULC changes in individual continents and countries**

In Asia, the amount of land that converted from bare to agriculture was the largest among all continents, particularly in Kazakhstan^56^. The area of agricultural land lost in Asia was the largest among all continents, larger than the size of Thailand, which took place mainly through conversion to urban areas in China^57^ and India^58^. Our findings show that over 31% of agricultural land lost in Asia converted to urban areas. Furthermore, Asia experienced the largest gain in urban area worldwide comprising 45% of the global gain, particularly in China^59^ and India^60^, and our findings are in accordance with Dou and Kuang^61^. In addition to China and India, other nations including Pakistan^62^, Uzbekistan^63^, Bangladesh^64^, Vietnam^65^, and Thailand^66^ have experienced high percentages of urban expansion not only in Asia but also at the global scale. The amount of forest cover lost in Asia was the second largest of all continents. Large areas have experienced deforestation across Southeast Asia including Indonesia and Malaysia due to agricultural expansion via palm oil and rubber plantations^67,68^ and China due to urban expansion^34^. However, the highest percentages of forest loss were found in Cambodia^69^ due to the expansion of cassava and rice plantations and Vietnam^70^ due to the expansion of commodity crops including coffee and tea. On the other hand, there was a noticeable trend of afforestation recently in China^71^. Our forest cover findings are in line with those of Duan and Tan^30^.

In Africa, several countries (e.g., Tanzania^6^ and Zambia^72^), witnessed a large agricultural expansion, mainly at the expense of forests and natural vegetation. We found that forest cover converting to agriculture was the second largest continental transition. Furthermore, considerable agricultural land was lost due to urban expansion, including in Nigeria^73^ and Egypt^4^, and we found that 51% of the gain in urban area in Africa was at the expense of agriculture^74^. The reduction of natural vegetation cover in Africa was the largest of all continents and the net natural vegetation cover lost in Africa contributed an astonishing 91% of the global net loss. Although the overall net change in forest cover was a net gain, there were considerable high deforestation rates in individual countries including Malawi^75^ and Liberia^76^, mainly due to the expansion of commercial crops such as tobacco in the former and rubber in the latter. Recently, several concerns have been raised regarding the expansion of commodity crops for export to lucrative markets in Sub-Saharan Africa^77^.

In South America, agricultural land saw a significant net gain, equivalent to the size of Japan. Consequently, significant forest cover decline occurred, in which 60% of the gross decline transitioned to agriculture. The net forest cover decline was larger than the size of Paraguay. Our forest cover findings were in line with the FAO^46^ and Duan and Tan^30^. This significant area of forest cover lost in South America is clear evidence of the ongoing and continual deforestation processes within one of the most vital places on Earth, the Amazon rainforest^78^. Soybean production is a major contributor to deforestation processes occurring in South America, with the largest amount of forest cover lost in Brazil^79^. Furthermore, High percentage rates of deforestation were found in Argentina, Paraguay, and Bolivia due to cattle ranching and pasture expansion as well as soybean plantations^80^.

In North America, the transitions between forest cover and bare land were the largest among all continents, and they were located mainly in Canada^81^. This can be attributed to boreal forest loss due to fires^82^, insect infections and logging^83^. Furthermore, the areas of forest cover and natural vegetation transitioning to urban were the largest among all continents, and they were located mainly in the USA^84^. Guatemala^85^ and Nicaragua^86^ experienced high deforestation rates, not only in the Americas but also at the global scale. This could be attributed to the expansion of palm oil plantations in the former nation and coffee in the latter. Another factor causing this issue in Central America was the illegal cattle ranching expansion in relation to illegal cocaine trafficking^85^.

In Europe, agricultural abandonment is a common issue, and it is a major contributor to the continental decline observed, particularly in Eastern Europe^87^. Europe has also witnessed significant agricultural land loss due to urban expansion^88^, and we found that this particular LULC transition was larger in Europe than all other continents. The amount of agricultural land lost to urban growth was equivalent to the size of the Czech Republic. However, recently, there was a trend in Eastern Europe to recultivate abandoned agricultural land^89^. Urban gain in Europe was the second largest of all continents, covering an area equivalent to the size of Portugal. Our observations of the increasing forest cover in Europe were in line with those of the FAO^46^.

In Oceania, the transitions between natural vegetation and bare land were the largest among all continents by far, and this may be attributed to climatic variability, particularly in Australia^90^. For example, alternation of extended periods of drought and intermittent rainfall are strong drivers of sporadic growth phases in vegetation in this region in arid and semi-arid zones.

Generally, it is noted that the total areas of LULC change in developed regions, including Europe and North America, were much smaller than the corresponding area of changes in developing regions, including Asia and Africa. This can be attributed to the implementation of effective policies and sustainable management strategies in developed regions, and more unrestrained LULC management in developing regions^91^.

**Supplementary References**

4. Radwan, T. M., Blackburn, G. A., Whyatt, J. D. & Atkinson, P. M. Dramatic loss of agricultural land due to urban expansion threatens food security in the Nile Delta, Egypt. Remote Sens. 11, 332 (2019).

6. Nzunda, E. F. & Midtgaard, F. Deforestation and loss of bushland and grassland primarily due to expansion of cultivation in mainland Tanzania (1995–2010). J. Sustain. For. 38, 509–525 (2019).

30. Duan, Q. & Tan, M. Spatial and temporal variations of forest cover in developing countries. Sustain. 11, 1517 (2019).

34. Ji, Y. et al. Unbalanced forest displacement across the coastal urban groups of eastern China in recent decades. Sci. Total Environ. 705, 135900 (2020).

36. Environmental Systems Research Institute (ESRI). ArcGIS Desktop 10.5; ESRI: Redlands, CA, USA. (2016).

46. FAO. Global Forest Resources Assessment 2015 Desk reference. Food and Agriculture Organization of the United Nations, Rome. (2015).

56. Meyfroidt, P., Schierhorn, F., Prishchepov, A. V., Müller, D. & Kuemmerle, T. Drivers, constraints and trade-offs associated with recultivating abandoned cropland in Russia, Ukraine and Kazakhstan. *Glob. Environ. Chang.* **37**, 1–15 (2016).

57. Cui, Y. *et al.* Accelerating cities in an unsustainable landscape: Urban expansion and cropland occupation in China, 1990-2030. *Sustain.* **11**, 2283 (2019).

58. Tang, J. & Di, L. Past and future trajectories of farmland loss due to rapid urbanization using Landsat imagery and the Markov-CA model: A case study of Delhi, India. *Remote Sens.* **11**, 180 (2019).

59. Wu, W., Zhao, S., Zhu, C. & Jiang, J. A comparative study of urban expansion in Beijing, Tianjin and Shijiazhuang over the past three decades. *Landsc. Urban Plan.* **134**, 93–106 (2015).

60. Sahana, M., Hong, H. & Sajjad, H. Analyzing urban spatial patterns and trend of urban growth using urban sprawl matrix: A study on Kolkata urban agglomeration, India. *Sci. Total Environ.* **628**–**629**, 1557–1566 (2018)

61. Dou, Y. & Kuang, W. A comparative analysis of urban impervious surface and green space and their dynamics among 318 different size cities in China in the past 25 years. *Sci. Total Environ.* **706**, 135828 (2020).

62. Bhatti, S. S., Tripathi, N. K., Nitivattananon, V., Rana, I. A. & Mozumder, C. A multi-scale modeling approach for simulating urbanization in a metropolitan region. Habitat Int. **50**, 354–365 (2015).

63. Conrad, C. *et al.* Measuring rural settlement expansion in Uzbekistan using remote sensing to support spatial planning. *Appl. Geogr.* **62**, 29–43 (2015).

64. Hassan, M. M. & Southworth, J. Analyzing land cover change and urban growth trajectories of the mega-urban region of Dhaka using remotely sensed data and an ensemble classifier. *Sustain.* **10**, 1–24 (2017).

65. Vu, T. T., Thy, P. T. M. & Nguyen, L. Đ. Multiscale remote sensing of urbanization in Ho Chi Minh city, Vietnam - A focused study of the south. *Appl. Geogr.* **92**, 168–181 (2018).

66. Estoque, R. C. & Murayama, Y. Intensity and spatial pattern of urban land changes in the megacities of Southeast Asia. *Land use policy* **48**, 213–222 (2015).

67. Zeng, Z. *et al.* Highland cropland expansion and forest loss in Southeast Asia in the twenty-first century. *Nat. Geosci.* **11**, 556–562 (2018).

68. Estoque, R. C. *et al.* The future of Southeast Asia’s forests. *Nat. Commun.* **10**, 1–12 (2019).

69. Kong, R. *et al.* Understanding the drivers of deforestation and agricultural transformations in the Northwestern uplands of Cambodia. *Appl. Geogr.* **102**, 84–98 (2019).

70. Meyfroidt, P., Vu, T. P. & Hoang, V. A. Trajectories of deforestation, coffee expansion and displacement of shifting cultivation in the Central Highlands of Vietnam. *Glob. Environ. Chang.* **23**, 1187–1198 (2013).

71. Piao, S. *et al.* Detection and attribution of vegetation greening trend in China over the last 30 years. *Glob. Chang. Biol.* **21**, 1601–1609 (2015).

72. Phiri, D., Morgenroth, J. & Xu, C. Four decades of land cover and forest connectivity study in Zambia—An object-based image analysis approach. *Int. J. Appl. Earth Obs. Geoinf.* **79**, 97–109 (2019).

73. Nkeki, F. N. Spatio-temporal analysis of land use transition and urban growth characterization in Benin metropolitan region, Nigeria. *Remote Sens. Appl. Soc. Environ.* **4**, 119–137 (2016).

74. Güneralp, B., Lwasa, S., Masundire, H., Parnell, S. & Seto, K. C. Urbanization in Africa: challenges and opportunities for conservation. *Environ. Res. Lett.* **13**, 15002 (2017).

75. Ngwira, S. & Watanabe, T. An Analysis of the Causes of Deforestation in Malawi: A Case of Mwazisi. *Land* **8**, 48 (2019).

76. Enaruvbe, G. O., Keculah, K. M., Atedhor, G. O. & Osewole, A. O. Armed conflict and mining induced land-use transition in northern Nimba County, Liberia. *Glob. Ecol. Conserv.* **17**, e00597 (2019).

77. Ordway, E. M., Asner, G. P. & Lambin, E. F. Deforestation risk due to commodity crop expansion in sub-Saharan Africa. *Environ. Res. Lett.* **12**, (2017).

78. Lu, D., Li, G., Moran, E. & Hetrick, S. Spatiotemporal analysis of land-use and land-cover change in the Brazilian Amazon. *Int. J. Remote Sens.* **34**, 5953–5978 (2013).

79. Santos, C. A. G., do Nascimento, T. V. M. & da Silva, R. M. Analysis of forest cover changes and trends in the Brazilian semiarid region between 2000 and 2018. *Environ. Earth Sci.* **79**, 1–20 (2020).

80. Fehlenberg, V. *et al.* The role of soybean production as an underlying driver of deforestation in the South American Chaco. *Glob. Environ. Chang.* **45**, 24–34 (2017).

81. Fitzsimmons, M. Effects of deforestation and reforestation on landscape spatial structure in boreal Saskatchewan, Canada. *For. Ecol. Manage.* **174**, 577–592 (2003).

82. Hicke, J. A. *et al.* Postfire response of North American boreal forest net primary productivity analyzed with satellite observations. *Glob. Chang. Biol.* **9**, 1145–1157 (2003).

83. Kukavskaya, E. A. *et al.* Influence of logging on the effects of wildfire in Siberia. *Environ. Res. Lett.* **8**, (2013).

84. Zhang, C. *et al.* Impacts of urbanization on carbon balance in terrestrial ecosystems of the Southern United States. *Environ. Pollut.* **164**, 89–101 (2012).

85. Devine, J. A., Currit, N., Reygadas, Y., Liller, L. I. & Allen, G. Drug trafficking, cattle ranching and Land use and Land cover change in Guatemala’s Maya Biosphere Reserve. *Land use policy* **95**, 104578 (2020).

86. Tobar-López, D., Bonin, M., Andrade, H. J., Pulido, A. & Ibrahim, M. Deforestation processes in the livestock territory of La Vía Láctea, Matagalpa, Nicaragua. *J. Land Use Sci.* **14**, 225–241 (2019).

87. Estel, S. *et al.* Mapping farmland abandonment and recultivation across Europe using MODIS NDVI time series. *Remote Sens. Environ.* **163**, 312–325 (2015).

88. Salvati, L., Zambon, I., Chelli, F. M. & Serra, P. Do spatial patterns of urbanization and land consumption reflect different socioeconomic contexts in Europe? *Sci. Total Environ.* **625**, 722–730 (2018).

89. Smaliychuk, A. *et al.* Recultivation of abandoned agricultural lands in Ukraine: Patterns and drivers. *Glob. Environ. Chang.* **38**, 70–81 (2016).

90. Long, X. *et al.* Response of vegetation cover to climate variability in protected and grazed arid rangelands of South Australia. *J. Arid Environ.* **161**, 64–71 (2019).

91. United Nations. *Human Development Indices and Indicators. Statistical Update*. (2018).
